# Introducing AOD 4: A dataset for air borne object detection

**DOI:** 10.1016/j.dib.2024.110801

**Published:** 2024-08-06

**Authors:** Vama Soni, Dhruval Shah, Jeel Joshi, Shilpa Gite, Biswajeet Pradhan, Abdullah Alamri

**Affiliations:** a Department of Computer Science and Engineering, Devang Patel Institute of Advance of Technology and Research (DEPSTAR), Charotar University of Science and Technology (CHARUSAT), Changa, Gujarat, 388421, India; b Department of Information Technology, Devang Patel Institute of Advance of Technology and Research (DEPSTAR), Charotar University of Science and Technology (CHARUSAT), Changa, Gujarat, 388421, India; c Department of Artificial Intelligence and Machine Learning, Symbiosis Institute of Technology, Symbiosis Centre of Applied Artificial Intelligence (SCAAI), Symbiosis International (Deemed) University, Pune, 412115, India; d Centre for Advanced Modelling and Geospatial Information Systems (CAMGIS), School of Civil and Environmental Engineering, University of Technology Sydney, NSW 2007, Australia; e Department of Geology and Geophysics, College of Science, King Saud University, Riyadh, Saudi Arabia

**Keywords:** Airplanes, Helicopter, Drones, Bird, Complex environment

## Abstract

This paper introduces an airborne object dataset comprising 22,516 images categorizing four classes of airborne objects: airplanes, helicopters, drones, and birds. The dataset was compiled from YouTube-8 M, Anti-UAV, and Ahmed Mohsen's dataset hosted on Roboflow. Videos were sourced from the first two platforms and converted into individual frames, whereas the latter dataset already consisted of images. Following collection, the dataset underwent labelling and annotation processes utilizing Roboflow's annotation tool, resulting in 7,900 annotations per class. Researchers can leverage this dataset to develop and refine algorithms for airborne object detection and tracking, with potential applications spanning military surveillance, border security, and public safety.

Specifications Table

**Table:** 

Subject	Computer Vision and Pattern Recognition
Specific subject area	Airborne Object Detection
Type of data	Raw images with label files (.txt, .json, .xml, .pbtxt, .tfrecord)
Data collection	Data were collected from three sources: YouTube-8 M [1], Anti-UAV [2], and Ahmed Mohsen's dataset [3]. The collection was done by extracting frames from videos, manually annotating the images into four classes and then resizing the images into 1024 × 1024 pixels.
Data source location	YouTube-8M: https://research.google.com/youtube8m/ Anti-UAV: https://github.com/ucas-vg/Anti-UAV Ahmed Mohsen's dataset: https://universe.roboflow.com/ahmedmohsen/drone-detection-new-peksv
Data accessibility	Repository name: **mendeley.com** Data identification number: DOI: 10.17632/cd5z895tr2.1 Direct URL to data: https://data.mendeley.com/datasets/cd5z895tr2/1
Related research article	Not applicable

## 1 Value of the Data

• The dataset serves as a comprehensive resource for researchers in aerial object detection and computer vision. Its diverse collection of images and balanced labeling facilitates the training and evaluation of object detection models across multiple classes, including airplanes, helicopters, drones, and birds.
• Researchers can utilize the AOD-4 dataset to create and refine detection algorithms for real-world applications like aerial surveillance and security, improved search and rescue operations, and optimized traffic and air-space management.
• Along with object detection and classification, the dataset can be used for scene understanding and data augmentation.
• This dataset can function as a foundation for the creation of more expansive datasets. The existing class labels and annotations for airplanes, drones, birds, and helicopters provide a valuable reference point. Researchers can leverage this information to manually annotate images for new classes like balloons or specific scenarios, for example, airplanes at night, to enrich the dataset's scope.
• The dataset serves as a benchmark for evaluating detection model performance, aiding in assessing algorithm effectiveness and generalization capabilities.
• The dataset can be instrumental in educational purposes, enriching learning experiences for students and practitioners in understanding real-world scenarios.
• Its broad representation allows cross-domain research exploration, opening doors to novel applications beyond traditional aerial object detection by offering diverse images with varied objects, backgrounds, and attributes.

## 2 Background

Object detection in aerial imagery faces challenges due to limitations in existing datasets, which either focus on a single object class or include multiple objects with significant class imbalances. A comprehensive survey revealed gaps in object diversity, environmental variety, and representation of different object sizes and positions. Additionally, most datasets focus on medium-sized objects centered in the frame, neglecting the need to detect objects of varying sizes and positions. Furthermore, variable image resolutions lead to inconsistencies in data quality and training effectiveness. These shortcomings hinder the development of robust, generalizable detection algorithms for real-world scenarios.

To address these issues, the AOD-4 dataset was compiled, aiming to provide a balanced and comprehensive resource for advancing aerial object detection by including multiple object classes (airplanes, helicopters, birds, and drones), diverse environmental conditions, objects of varying sizes and positions, and both single and multiple object instances. By offering standardized image resolution and rich annotations, AOD-4 seeks to overcome the limitations of existing datasets and provide a solid foundation for developing and evaluating more accurate and versatile aerial object detection models, ultimately enhancing performance in real-world applications.

## 3 Data Description

The AOD-4 dataset, which includes 22,516 images and 31,600 annotations in four different classes (airplane, helicopter, drone, and bird), is an extensive collection of aerial imagery carefully annotated to aid with object detection endeavours. The dataset was derived from diverse sources, such as the YouTube-8 M dataset, the Anti-UAV dataset, and Ahmed Mohsen's dataset from Roboflow. The thoughtful curation of the dataset ensures a wide representation of airborne objects, which enhances its relevance to real-world situations. The dataset was divided into 70 % training (15,761 images), 20 % testing (4514 images), and 10 % validation (2241 images). The images were annotated on Roboflow [4], striving for balanced labelling across all classes to mitigate biases [5]. The train, test and validate split across all classes is mentioned in Table 1.

**Table 1:** Categorization of the images within the dataset.

Classes	Images			Annotations		
	Train	Valid	Test	Train	Valid	Test
Airplane	3885	1119	551	5508	1625	767
Helicopter	4032	1158	574	5528	1585	787
Drone	5199	1485	742	5502	1602	796
Bird	2255	638	320	5522	1557	821
Null	382	117	51	0	0	0

**Fig. 1:**
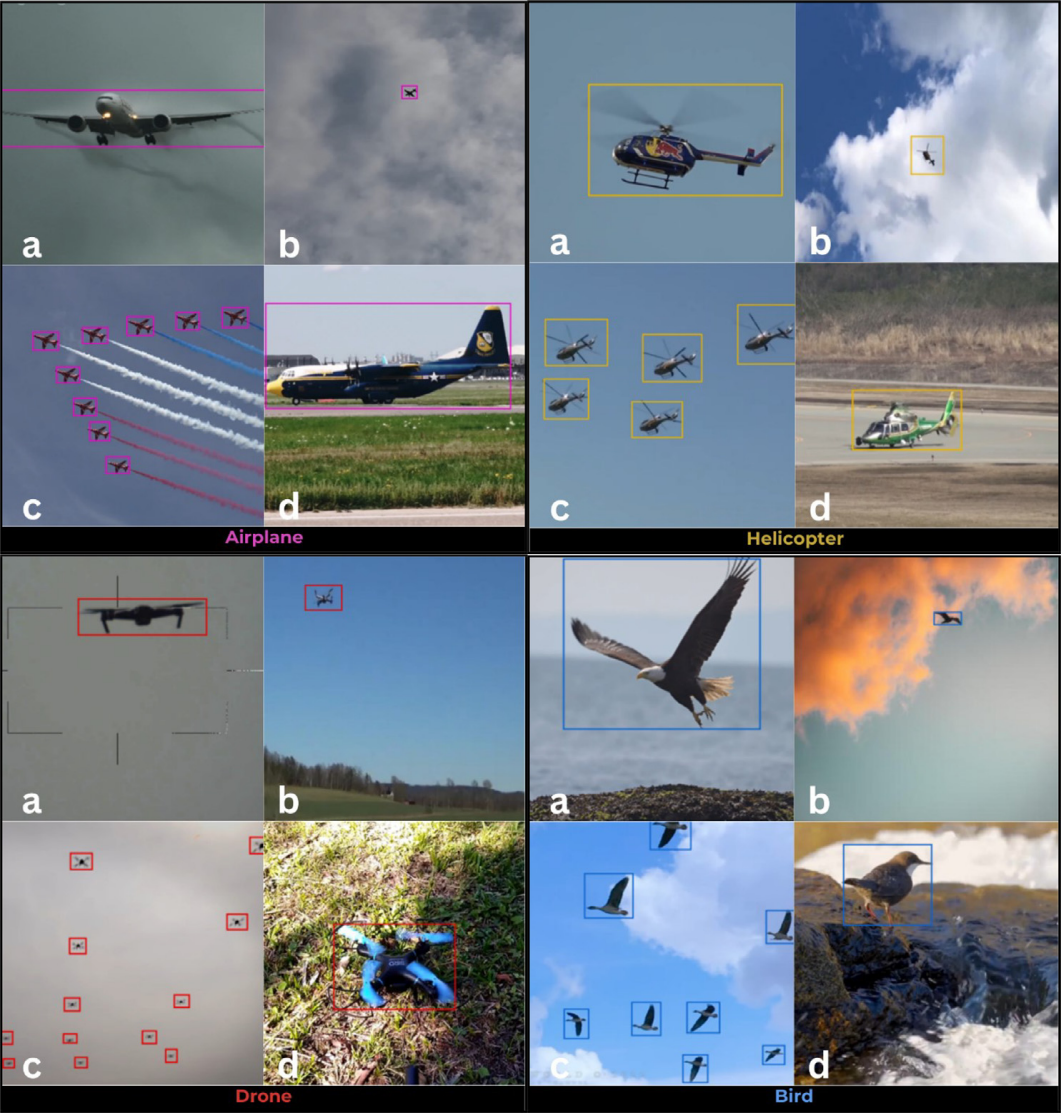
Sample images of four airborne classes - airplane, helicopter, drone, and bird from the AOD-4 dataset. Fig. 1a Large objects captured at close range, highlighting the detailed features of each object. Fig. 1b Small-sized objects captured from a distance, demonstrating the appearance of these objects when observed from afar. Fig. 1c Multiple objects captured illustrating the variation and group behaviour within each class. Fig. 1d Still Objects.

The dataset's richness includes a variety of backgrounds, including clouds, trees, skies, mountains, urban landscapes, and unfavourable weather conditions like rain and snow. Additionally, the dataset includes images with null annotations (no objects), enabling the detector to learn about the absence of relevant objects, thus reducing false positives. The dataset exhibits flexibility by including images showcasing single and multiple objects, ensuring adaptability to various analytical requirements, as shown in Fig. 1. Consistency in image dimensions (1024 × 1024 pixels) allows for easy analysis, and object characteristics like sizes, colors, and angles give a detailed representation of airborne objects. The characteristics and usefulness of the AOD-4 dataset are highlighted in Table 2.

**Table 2:** Dataset characteristics table.

Characteristic	Description
**Classes**	Since most existing datasets focus only on a limited number of airborne categories, we created a dataset comprising four classes—airplanes, helicopters, drones, and birds
**Number of Objects per class**	There are a total of 7900 objects per class + 550 null annotations, making the dataset well-balanced. This results in a total of 22,516 images.
**Variety of Environmental conditions**	Having images with varying backgrounds is extremely important for creating models that are robust to changing environments. The AOD-4 dataset consists of images with sky, cloudy, terrestrial backgrounds, as well as nighttime, rainy, and snowy conditions.
**Number of objects per frame**	The dataset consists of images with a single object and multiple objects in the frame. Thus, the dataset can easily be used for both single-object as well as multiple-object detection. On average, there are 1.4 objects per image.
**Size of objects**	Covering a wide range of object sizes results in good model performance and robustness. Therefore, the dataset includes objects of varying sizes, from tiny to medium-sized and large.
**Resolution**	The images of varying sizes are all resized to be consistent at 1024 × 1021 pixels.
**Position of objects in the frame**	The objects in the image are present all across the frame

## 4 Experimental Design, Materials and Methods

### 4.1 Analysis of existing datasets

To address limitations in existing airborne object detection (AOD) datasets, we conducted an extensive survey. The results from the study, as discussed in the Background section of this paper, highlight the need of a more balanced, comprehensive, and standardized dataset, like AOD-4, to address the existing gaps by enhancing class diversity, environmental variety, object size and positioning considerations, and image resolution.

### 4.2 Data collection

The AOD-4 dataset was meticulously curated from three different sources, namely the Anti-UAV dataset, the YouTube-8 M dataset, and Ahmed Mohsen's dataset, to address existing gaps in aerial object detection datasets. The Anti-UAV dataset was utilized for the extensive collection of drone images, focusing on RGB formats. Images of all four classes - airplanes, helicopters, drones, and birds were extracted from YouTube videos available on the YouTube-8 M dataset. This source contributed significantly to the variability of the dataset. Ahmed Mohsen's dataset from Roboflow also provided a rich pool of images of all four classes. This collection included a range of scenarios such as distant objects, occlusions, environmental variations, and dynamic movements, ensuring a diverse representation of object sizes and positions within frames. The balanced approach in the number of objects per class mitigates biases and enhances the dataset's utility in training robust detection models. Environmental diversity was also a key consideration, with images featuring backgrounds such as skies, clouds, mountains, terrestrial landscapes such as grasslands, forests, deserts, and challenging weather conditions like rain and snow. Additionally, the dataset includes instances with both single and multiple objects per frame, accommodating different object sizes and their positions within the image. Finally, to simulate real-world scenarios, we deliberately introduced null images.

### 4.3 Annotations

Our annotation process leveraged Roboflow, a comprehensive data annotation platform, to ensure consistency and efficiency. We incorporated high-quality images from the Anti-UAV and Ahmed Mohsen's datasets on Roboflow. These pre-annotated images were carefully handpicked to maintain data integrity. To broaden the dataset's scope, we extracted video frames from the YouTube-8 M dataset and uploaded them to Roboflow for manual annotation. Converting videos into individual frames facilitated a more granular labelling process. We meticulously annotated each image into four distinct classes: airplane, helicopter, drone, and bird.

### 4.4 Export

**Fig. 2:**
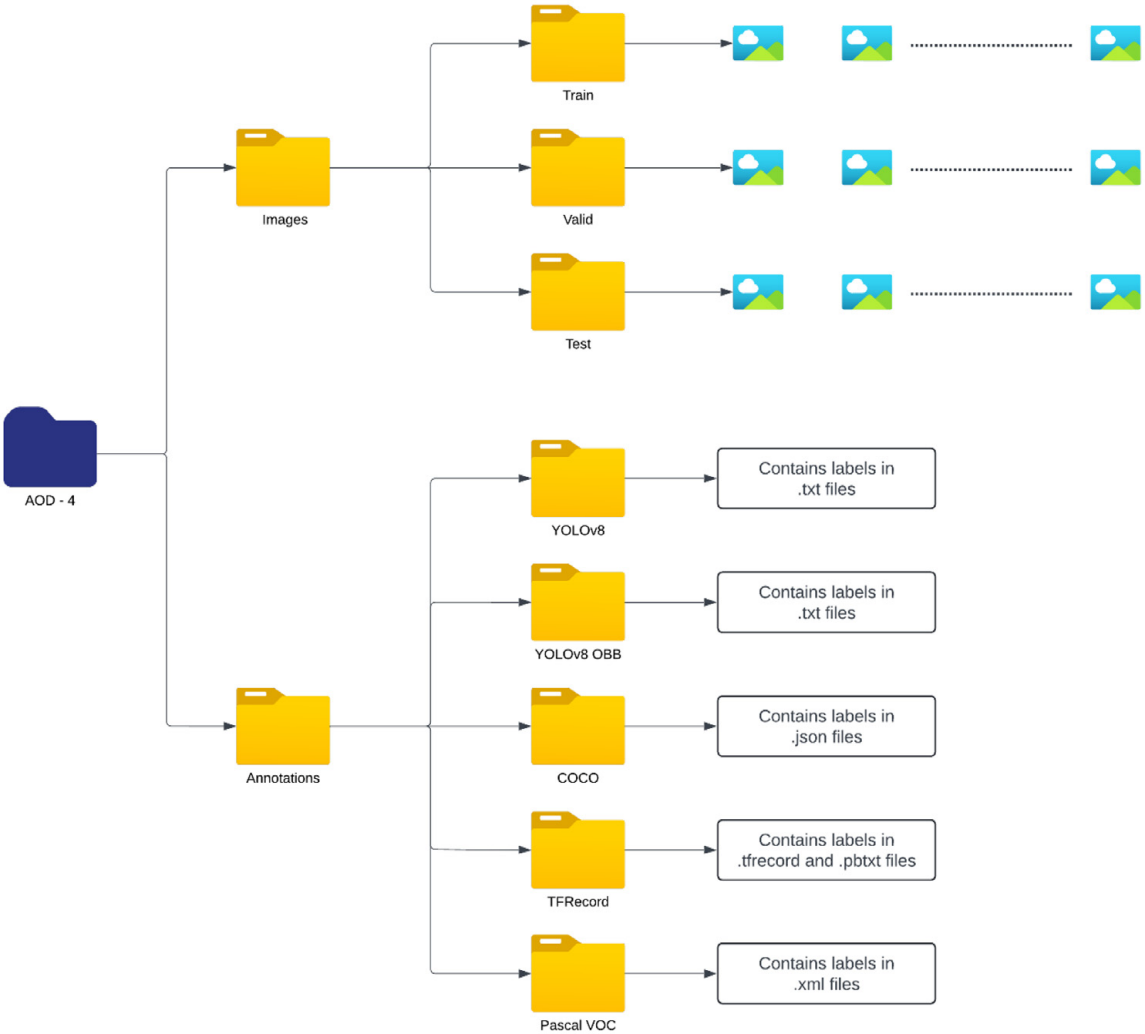
Dataset file structure.

Upon completion of the annotation process, the entire dataset was uniformly resized to a consistent resolution of 1024 × 1024 pixels to maintain data quality and consistency. The annotations were then exported in multiple formats, including YOLOv8, YOLOv8 OBB, COCO, PASCAL VOC, and TF Record as shown in Fig. 2. This multi-format export capability ensures that the AOD-4 dataset is compatible with a wide range of object detection frameworks and tools. This facilitates ease of use for various research and development projects.

## Limitations

The current dataset, while offering a diverse representation of airborne objects, exhibits several limitations that may impact the performance and generalizability of object detection models.

• **Limited Object Class Coverage:** The dataset primarily focuses on four primary object classes: airplanes, drones, helicopters, and birds. While this encompasses a broad spectrum of airborne entities, it excludes crucial categories such as balloons (both hot-air and weather), airships, and parachutes. Expanding the object vocabulary to include these classes is essential for training object detection models capable of recognizing a wider range of airborne objects.
• **Background Diversity Imbalance:** While the dataset includes images captured across various conditions, there needs to be more consistent background diversity across different object classes. For example, while helicopter images are captured in a wide range of environments, including snowy conditions, images of airplanes, drones, and birds predominantly exclude snowy backgrounds. This imbalance in background representation may limit a model's ability to generalize to real-world situations with diverse and complex backgrounds. Controlled data collection by adding images from diverse environmental conditions can resolve this background imbalance.
• **Intra-class Variability Constraints:** The dataset exhibits limited intra-class diversity, restricting a model's ability to learn subtle variations within each object class. Incorporating a broader range of airplane types (e.g., military, commercial), advanced drone models, helicopter configurations (e.g., tandem rotor), and bird species would significantly enrich the dataset and improve model performance.
• **Insufficient Low-Light and Nighttime Imagery:** The dataset primarily comprises daytime imagery, limiting its applicability to real-world scenarios with varying lighting conditions. To enhance model robustness and adaptability, the inclusion of low-light and nighttime images, as well as infrared imagery, is crucial.

By addressing these limitations through dataset expansion and augmentation, the potential of object detection models can be significantly enhanced, leading to improved performance and broader applicability in real-world applications.

## Ethics Statement

The authors have confirmed that this study does not involve human subjects, animal experiments, or any data collected from social media platforms and follows the ethical requirement for publication in Data in Brief.

## CRediT Author Statement

**Vama Soni:** Conceptualization, Investigation, Data curation, Writing – original draft, Visualization; **Dhruval Shah**: Conceptualization, Investigation, Data curation, Writing – original draft, Visualization; **Jeel Joshi**: Conceptualization, Investigation, Data curation, Writing – original draft, Visualization; **Shilpa Gite:** Writing - Review & Editing, Supervision; **Biswajeet Pradhan:** Writing - Review & Editing, Supervision, Funding, Resources; **Abdullah Alamri:** Writing - Review & Editing, Funding.

## Data Availability

Introducing AOD 4: A Dataset for Air Borne Object Detection (Original data) (Mendeley Data).
